# Incubation and interactivity in insight problem solving

**DOI:** 10.1007/s00426-018-0992-9

**Published:** 2018-02-26

**Authors:** Niyat Henok, Frédéric Vallée-Tourangeau, Gaëlle Vallée-Tourangeau

**Affiliations:** 1 Department of Management, Kingston University Business School, Kingston-upon-Thames, KT2 7LB UK; 2 Department of Psychology, Kingston University, Kingston-upon-Thames, KT1 2EE UK

## Abstract

Insight is commonly viewed as originating from the restructuring of a mental representation. Distributed cognition frameworks such as the Systemic Thinking Model (SysTM, Vallée-Tourangeau and Vallée-Tourangeau, Cognition beyond the brain: interactivity and human thinking, pp 133–154, 2017), however, assumes that information processing can be transformed when it is distributed across mental and material resources. The experiments reported here investigated whether interactivity enhanced incubation effects with the cheap necklace problem. Participants attempted to solve the problem in a low-interactivity condition with pen and paper or in a high-interactivity condition with a set of metal chains. Performance was substantially better in a task environment that fostered a higher degree of interactivity at Time 1. There was evidence of an incubation effect as participants significantly improved in performance after a 2-week gap, particularly in the high-interactivity condition. Experiment 2 showed that the context within which people can enact their thinking following incubation is key to improve problem-solving performance. When the problem presentation changed after a 2-week gap (low interactivity to high interactivity or high interactivity to low interactivity), performance only improved for those who worked on a highly interactive task at Time 2. Taken together, these findings underscore the importance of adopting a systemic perspective when investigating incubation effects in problem solving.

## Incubation and interactivity in insight problem solving

In general day-to-day activities, problems range in nature and complexity. Different strategies are used to solve these problems based on previous experience, seeking help from others, utilising information and tools immediately available, and so on. Traditionally, psychologists classify problems into two broad categories: transformation and insight. Transformation problems are well defined with a clear specified goal, such as the Tower of Hanoi. The problem space identifies the sequence of steps that transforms the problem from the initial state to the goal state. By contrast, insight problems are less well defined, and are formulated in a manner that derails participants’ ability to anticipate a path to solution.

**Fig. 1:**
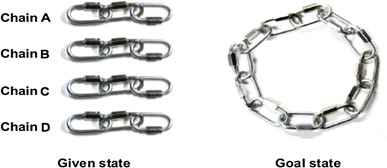
Given and goal states in the cheap necklace problem

The cheap necklace problem (CNP) is one such insight problem. Introduced by Silveira (1971), the CNP is a difficult problem with a solution rate often less than 10% (Fioratou & Cowley, 2009; Fioratou, Flin, & Glavin, 2010; Silveira, 1971). In its standard form, participants are presented with a diagram of four chains each consisting of three links, alongside a diagram of a complete 12-link necklace. Figure 1 presents a slightly modified version wherein actual chains are shown in a photograph rather than illustrated with a schematic diagram. Participants are asked to reconstruct the necklace by connecting the four separate chains at a cost: namely, 2¢ to open a link, 3¢ to close a link, with a maximum spending of 15¢. It is not possible to solve this task by simply connecting the ends of each chain, as this will exceed the 15¢ allowed. Joining the ends of each chain suggests optimal progress towards the solution as the initial move of joining two chains together only cost 5¢, yet makes half of the necklace. This path, however, leads to a dead end as it will eventually cost 20¢. Although joining the ends of the chains does not work, participants have been observed to persevere with this strategy (Chu, Dewald & Chronicle, 2007). The solution requires all the links in one chain to be opened at a cost of 6¢, then connecting the remaining three chains together using those open links, at a cost of 9¢. The solution to break up the chain with the aim of making a complete necklace is counterintuitive.

### Incubation

When struggling to solve a problem, it may pay off to leave it aside for some time before attempting to discover a solution. In problem-solving research, the initial period of unsuccessful labour is sometimes referred as a ‘preparation’ phase and the interpolated break period the ‘incubation’ phase (Wallas, 1926). There is evidence that taking a break from the CNP after struggling to solve it results in higher solution rates later. Silveira (1971, Experiment 2) sought to establish whether the difficult CNP would be made easier when delays were placed between an initial intense period of working on the problem and a subsequent attempt at completing it. In her study, participants were shown the CNP on a sheet of paper. They were told they were free to make notes on the paper while they thought about the problem. They were also made aware that they would be interrupted, but were not told when. Thirteen minutes after reaching an impasse (for example, closing a circle of chains and realising they would go over budget), participants were interrupted to complete a short questionnaire about their performance. A control group could resume work on the problem as soon as the questionnaire was completed. The experimental group was given a 4-h break before resuming work on the CNP. After this incubation period, they were given the same sheet they had previously been working with and continued searching for a solution. Participants across all conditions performed well in the task, with successful performance at 38 and 81%, respectively. Overall successful performance was very high in this study in comparison to subsequent studies on the CNP, which may be explained by the extended period of time people were allowed to work on the problem (González-Vallejo, Lassiter, Bellezza, & Lindberg, 2008).

Sio and Ormerod’s (2009) meta-analysis examined incubation effects for three types of problems, creative (e.g. unusual uses test), visual insight problems (of which the CNP is an example, see Sio & Ormerod, p. 113), and linguistic insight problems (e.g. remote associate problems). Their meta-analysis reported significant incubation effects for all three types of problems. The main moderator of incubation effects for visual insight problems was the length of the preparation phase. Thus, a stronger incubation effect is observed the longer a participant struggles to solve the problem before the interpolated break period.

Incubation in tasks such as the CNP may help problem solving through restructuring following impasse (MacGregor, Ormerod, & Chronicle, 2001). Restructuring is conceived as the reorganisation of a reasoner’s mental representation in a more productive form, enabling a more adequate use of the problem information to reach a solution (Sio & Ormerod, 2009). Restructuring can proceed through switching search strategies for possible moves (e.g. MacGregor et al., 2001), relaxing constraints on possible moves as well as decomposing familiar patterns of features or “chunks” (e.g. Knoblich, Ohlsson Haider & Rhenius, 1999); some of these mental processes may be unconscious rather than reflecting a conscious deliberate analysis of the problem elements. In addition, withdrawing attention from a problem that resists a solution (and its concomitant incorrect representation) can enhance solution rates once the problem is revisited (Segal, 2004, Sio & Ormerod, 2009). One never steps into the same idea twice (to adapt Ingold, 2014) and returning to the problem after a delay may help defuse incorrect assumptions and encourage a different perspective on the problem.

Breaking one of the four chains into three separate links is the breakthrough required to solve the CNP problem. The difficulty in solving this problem is often attributed to the (mental) representation of the small chains as tight perceptual chunks. From the perspective of Ohlsson’s (1992) Representational Change Theory, for instance, a solution is more likely achieved if the representation is restructured such that individuated links are segmented and hence can be decomposed. Alternatively, the Criterion for Satisfactory Progress theory (Chu et al., 2007) proposes that hints aimed at defusing the first obvious move—namely to connect two chains to form half the necklace—may help reasoners solve the problem. In a series of experiments, Chu et al. established that a mix of hints to prevent selecting the obvious first move as well as presenting one of the four chains as made up of three links each of a separate colour was most efficient in improving solution rates. These findings suggest that both perceptual chunking and criterion for satisfactory progress contribute to the impasse.

### Cognitive interactivity and the systemic thinking model (SysTM)

**Fig. 2:**
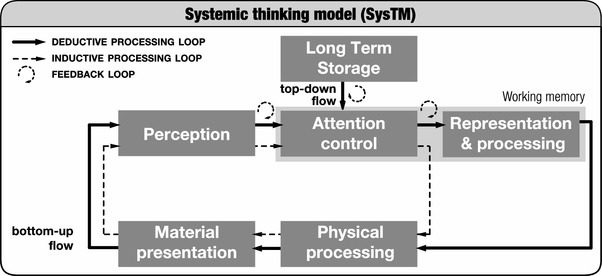
Systemic thinking model (SysTM, adapted from Vallée-Tourangeau & Vallée-Tourangeau, 2017)

Despite being worded as an active task where links are opened and closed, the CNP problem is traditionally offered as a pen and paper exercise. Yet, outside the psychologist’s laboratory, situations that require problem solving are rarely encountered in this manner. Anthropologists, cognitive archaeologists, human factors engineers, and designers are keenly aware of the role of artefacts in supporting and transforming thinking (e.g. Baber, Chemero, & Hall, 2017; Hutchins, 1995; Kirsh, 2013; Malafouris, 2014; Norman, 1993; Toon, 2011). Recently, the systemic thinking model (SysTM, Vallée-Tourangeau, Abadie, & Vallée-Tourangeau, 2015; Vallée-Tourangeau & Vallée-Tourangeau, 2017) was developed to account for interactivity, defined as the processes involved when thinking and problem solving take place in environments where people and things exert a reciprocal influence upon one another. (see Fig. 2).

SysTM highlights that when participants can act directly upon the material presentation of the information, thinking no longer results exclusively from the deductive processing of a mental representation (Rumelhart, Smolensky, McClelland, & Hinton, 1986). Rather, solutions to problems emerge from a situated agent–environment ecosystem: that is, from an agent situated in, interacting with, and being influenced by her physical environment. Extending our conception of cognitive activity beyond the remits of deductive mental processing allows introducing a different type of information processing, namely inductive processing, which may also transform, and possibly augment, thinking performance (Kirsh, 2017). Specifically, SysTM proposes that enabling the physical manipulation of one’s immediate environment supports the perception of “micro-affordances” (Ellis & Tucker, 2000), or possible actions within one’s environment that are perceived without being mediated by mental representations. As individuals act upon these perceived micro-affordances (e.g. by grasping a chain), they transform the perceptual input. This affordance-driven physical rearrangement allows cognition to proceed through an inductive processing loop where the physical manipulation of the material layout without a clear mental plan multiplies the opportunities to achieve different and potentially fruitful perceptual perspectives.

It follows that agent–environment ecosystems can be characterised by the level of interactivity they afford, that is, the extent to which the cognitive agent has the opportunity to transform the material instantiation of a cognitive task and, conversely, to be influenced by the task’s changing perceptual layout. One of the key predictions of SysTM is, therefore, that increasing the opportunities to physically manipulate information through highly interactive environments will increase the likelihood of successful problem solving.

Consistent with this view, increasing opportunities to interact with the problem material has been shown to increase performance with matchstick algebra problems (Weller, Villejoubert, & Vallée-Tourangeau, 2011) as well as the CNP (Fioratou & Cowley, 2009). To measure the impact of increasing interactivity in the CNP, Fioratou and Cowley (2009) compared two problem versions: an “abstract” paper-and-pencil version where participants were given a pen and could draw possible solutions and a “concrete (physical)” version where participants were presented with opening and closing metal chains to work on the problem. In line with the SysTM terminology, we will thereafter distinguish these versions by the level of interactivity they afford, and refer to them as “low-interactivity” and “high-interactivity” versions, respectively. With 10 min to reach a solution, fewer than 3% of the participants solved the problem with the low-interactivity version. This performance rate rose to 30% when participants were given the opportunity to interact with a physical model of the problem in the high-interactivity version. To summarise, when thinking can be reflected and prompted by changes in the world, the resulting perceptual change may highlight cues to new strategies, thus enabling better planning and efficiency in progressing towards a solution (Vallée-Tourangeau & Vallée-Tourangeau, 2017; Vallée-Tourangeau, 2014; Vallée-Tourangeau, Abadie, & Vallée-Tourangeau, 2015).

### Interactivity and incubation

Sio and Ormerod (2009) identify six possible moderators of incubation effects in problem solving, including length of the preparation phase and nature of the interpolated task (see pp. 95–97); however, they do not consider the interactive nature of the task environment. Rather, their perspective on problem solving is couched in cognitive terms aimed at eventually developing a ‘computational model of insight problem solving’ (p. 110), moderated by psychometric variables such as executive function and working memory capacity. Yet, as we mentioned above, another way to increase performance is to increase the interactivity afforded by the task presentation by giving participants the opportunity to manipulate actual chains. Participants in Silveira’s (1971) experiment did not have access to a material presentation of the cheap necklace. Thus, whether and how incubation effects may interact with higher degrees of task interactivity remains to be established.

Following the SysTM perspective, we can assume that working on a problem that offers limited opportunities for manipulating the task materials—as in the low-interactivity “pen-and-paper” version of the task—cognition will be constrained to follow deductive processing loops where actions (e.g. symbolic marks on the paper) proceed from a mentally represented plan. An unproductive task representation may lead to an impasse and restructuring may be hindered by the relative invariance of the informational input. Consider, by contrast, an information input which affords manipulation—as in the high-interactivity version mentioned above. Each manipulation of the metal chains has the potential to reconfigure the perceptual input. Akin to reasoners working on the low-interactivity version of the CNP, those engaged in interactive manipulation of metal chains in the high-interactivity version may restructure an unproductive task representation through planned hypothetico-deductive processing. The increased level of interactivity, however, also enables unplanned inductive processing. This does not only create more opportunities to identify a productive path to solution while they are working on the task, but it also fosters a richer, experiential and multisensory representation of the task information. This more dynamic and distributed representation may facilitate the discovery of new strategies and facilitate the relaxation of unproductive interpretations and constraints. Increased interactivity, we thus hypothesise, may favour productive restructuring when participants who failed to solve the problem at their first attempt may now be more likely to solve it on a second attempt after taking a break from working on the problem. In other words, an incubation effect may be more strongly manifested in a context that offers opportunities for action.

The SysTM perspective does not directly address whether incubation might be the product of largely deliberate conscious processing or unconscious processing—such as spread of activation or selective forgetting during the interpolated break. This is largely because the debate in its current form (e.g. Sio & Ormerod, 2009, pp. 94–95) is couched in computationalist terms: in those accounts, the issue is not whether insight is mediated by physical processing in one’s immediate environment, but rather whether it occurs through mental unconscious processes or mental conscious processes. In other words, the current debate is formulated in a manner that validates rather than challenges commitments to the traditional mentalist framework. This being noted, the SysTM perspective offers the opportunity to broaden the debate by exploring how inductive processing and unplanned physical actions contribute to insight. Thus, in addition to exploring the impact of interactivity on solving the CNP, the experiments reported here tested the prediction that participants who failed to solve the problem at Time 1 would be more likely to solve it after a delay when they worked on the problem in a high-interactivity condition compared to those who returned to work on the problem in a low-interactivity task environment.

Operationalising incubation under laboratory conditions often proceeds with an intervening task that encourages reasoners to abandon working on the primary solving task for a short period of time, usually a few minutes (e.g. Patrick, 1986; Segal, 2004; Silveira, 1971), although it is sometimes difficult to determine the exact length of the incubation period (Sio & Ormerod, 2009). Anecdotal examples of incubation in science and the arts, however, span much longer periods of time, weeks or months (Weisberg, 2014). We were thus interested in exploring the impact of an incubation period that spanned weeks rather than minutes. Although it may be more difficult to control the nature of the activities that participants engage in during that period, it offers a test that is much closer to incubation as it manifests outside the psychologist’s laboratory.

## Experiment 1

The aim of Experiment 1 was to examine whether interactivity would moderate incubation in the CNP task. Participants worked on the CNP either in a low- or high-interactivity condition. Based on previous findings, we expected performance to be influenced by the degree of interactivity afforded by the task environment: manipulating the physical elements of the problem should yield a higher solution rate than simulating moves mentally. Our main objective, however, was to test whether a higher degree of interactivity would promote a more pronounced incubation effect when participants returned to the problem after a substantial delay.

In addition to measuring solution rates, we also explored how solution latencies may be affected by increasing interactivity, as well as whether participants’ memory abilities may moderate the impact of increasing interactivity. There is increasing evidence to suggest that working memory is implicated in insight problem solving (e.g. Chudersky, 2014). However, tests and measures of working memory and problem-solving performance that inform correlational analyses usually proceed in task environments that afford little or no interactivity. Interacting with a physical model of the problem has been shown to functionally enhance a participant’s working memory resources when working on a mental arithmetic task, since the physical model stores information and cues actions that may be more difficult to rehearse and simulate mentally (Vallée-Tourangeau, Sirota, & Vallée-Tourangeau, 2016a). We were interested in determining the extent to which working memory was also implicated in performance on the CNP task, and whether its implication depended on the level of interactivity afforded by the task.

We also developed a long-term memory task based on the material reported in Roediger and Karpicke (2006); participants were presented with a short description of the Sun––qua celestial body––and were tested for their memory of that material at Time 1 and again at Time 2, computing a difference score to determine the rate of memory decline. This measure was designed to explore whether a long-term memory index discriminated between solvers and non-solvers at Time 2. This was exploratory because we were not in a position to make a specific prediction: On the one hand, better long-term memory skills may make it easier for participants to remember strategies employed at Time 1, but not whether these were successful or not. On the other hand, better memory skills may help participants remember more quickly what strategies were unsuccessful and hence support their dismissal.

## Experiment 1: method

### Participants

Sixty-three undergraduate and postgraduate psychology students (53 females) volunteered to participate in exchange for course credits (*M*_age_ = 22.05, *SD* = 4.05). All participants were naïve to the CNP prior to participation.

### Materials

A six-page problem pack was given to participants at both Times 1 and 2. The packs included an informed consent form, a short informative article about the Sun (only given at Time 1), the CNP with instructions and a set of four metal chains consisting of three links, which could be opened and closed by screwing the top of each link for participants in the high-interactivity condition. Low-interactivity participants were presented the task on a sheet of paper, including a picture of the chains, with the problem written at the top and lined space to work on a solution below.

### Design and procedure

This experiment employed a 2 × 2 mixed design, where the between-subjects factor was the level of interactivity (low interactivity vs. high interactivity) and time was a repeated measures factor (Time 1 vs. Time 2). At Time 1 participants initially attempted to solve the CNP in either a low interactivity or high interactivity, which they again attempted in the same interactivity condition at Time 2, 2 weeks later.

All testing was conducted individually in a quiet room. At Time 1, participants were given 5 min to read a short summary about the Sun that contained 30 separate ideas written in 256 words (taken from Roediger & Karpicke, 2006). They were then presented with the CNP and the instructions were read aloud by the researcher. The picture of the CNP (as illustrated in Fig. 1) was shown to participants in both conditions. In both interactivity conditions, participants had up to 30 min to complete the task, with the option to stop whenever they chose. Once they were ready to announce their answer to the problem, participants in both interactivity conditions were asked to write it down and the time taken to completion was noted. Participants were then given a recall sheet, and were given 5 min to recall in either full sentences or bullet points any information they could remember from the short article they read before attempting the CNP. Participants were then debriefed, but no feedback on performance was given. At this time, they were asked to return to the laboratory 2 weeks later and not to discuss or work on the CNP during that period.

Two weeks later, participants were given 5 min at the start of the session to note anything they could remember from the short description of the Sun presented at Time 1. Long-term memory performance was indexed by taking the difference in the number of distinct ideas recalled at Time 1 and Time 2: The greater the difference in recall, the sharper was the decline in memory performance at Time 2. They were then presented with the CNP and given 30 min to solve the problem. Once they noted their solution, the time taken to complete the task was noted. A computation span (C-Span) working memory task was then presented to the participants (adapted from Ashcraft & Kirk, 2001). In the C-Span, participants solved a series of simple arithmetic expressions. The series were composed of two to six expressions. At the end of each series, participants were asked to remember the second number for each expression presented in their order of presentation.

## Experiment 1: results

### CNP performance

#### Solution rates

Successful performance was classified using Silveira’s (1971) solution of deconstructing one chain into three links at a cost of 6¢, and using those three separate links to combine the remaining three chains into a single circle at a cost of 9¢. Solution rates are reported in Table 1: Twelve (or 43%) of the participants solved the problem in the high-interactivity condition at Time 1, while two (or 6%) solved the problem in the low-interactivity condition; the difference in solution rates was significant, *χ*^2^(1, *N* = 63) = 12.42, *p* < .001. At Time 2, all the participants who solved the problem at Time 1, in both interactivity conditions, offered a correct solution. Of the 16 participants who had not solved the problem in the high-interactivity condition at Time 1, 7 (or 44%) solved it at Time 2; of the 33 participants who were unable to solve the problem in the low-interactivity condition at Time 1, 5 (or 15%) solved it at Time 2; the difference was significant, *χ*^2^(1, *N* = 49) = 4.77, *p* = .029. Because one of the four cells of the contingency table had expected frequencies lower than five, we also conducted a Fisher exact test, which was significant, *p* = .040, and supported the initial chi square test.

**Table 1:** Solution frequencies in the low- and high-interactivity conditions at Times 1 and 2, along with solution latencies (in seconds), long-term memory performance (LTM; indexed as the difference in recall accuracy at Time 2), and working memory capacity (C-Span)

Time 1	Low interactivity		High interactivity	
	Yes	No	Yes	No
Freq	2	33	12	16
%	6%	94%	43%	57%
Latency to solution				
*M*	806.5		787.3	
* SD*	333.0		247.0	

Time 2	Low interactivity				High interactivity			
	Yes	No	Yes	No	Yes	No	Yes	No
Freq	2	0	5	28	12	0	7	9
%	100%	0%	15%	85%	100%	0%	44%	56%
Latency to solution								
*M*	227.5		371.4		315.3		496.9	
*SD*	108.2		227.5		155.0		218.9	
LTM index								
*M*	− 2.5		− 4.0	− 3.1	− 4.1		− 2.6	− 4.1
*SD*	0.7		1.9	2.1	2.1		1.6	1.8
C-Span								
*M*	31.5		16.0	11.6	22.0		24.3	16.1
*SD*	10.6		8.3	7.7	11.0		8.8	9.9

#### Solution latencies

Latency to solution were considerably lower at Time 2 compared to Time 1 for those participants who had solved the problem at Time 1 (see Table 1). In a 2 × 2 mixed ANOVA, the main effect of time was significant, *F*(1, 12) = 26.8, *p* < .001, but the main effect of level of interactivity, *F* < 1, and the interaction between time and interactivity, *F* < 1, were not. If the latencies for the participants who only solved the problem at Time 2 are compared with the latencies for those who solved the problem at Time 1, the main effect of time was significant, *F*(1, 21) = 11.1, *p* = .003, but the main effect of interactivity, *F* < 1, and the interaction between time and levels of interactivity, were not significant, *F*(1, 21) = 2.28, *p* = .146.

Of the unsuccessful participants in the low-interactivity condition, seven participants persevered for 30 min. In the high-interactivity condition, among the 16 unsuccessful participants, 3 worked on the problem for 30 min. However, a better measure of diligence might be offered by the time spent on the problem by those who gave up before the allocated 30 min. Participants who gave up working on the problem did so quicker in the low-interactivity (*M* = 521 s, *SD* = 472) than in the high-interactivity (*M* = 764 s, *SD* = 447) condition, a marginally significant difference, *t*(37) = − 1.99, *p* = .054. In light of the fact that the length of the preparation phase was identified as a significant moderator of incubation effects in Sio and Ormerod’s (2009) meta-analysis, time working on the problem for the unsuccessful participants at Time 1 might predict success at Time 2, independent of the level of interactivity. While participants who first solved the problem at Time 2 spent on average of four additional minutes working on the problem during Time 1 (*M* = 1029.0, *SD* = 632.8) compared to those who did not solve the problem at Time 2 (*M* = 787.5, *SD* = 571.1), this difference was not significant, *t*(47) = 1.24, *p* = .221.

### Long-term and working memory performance

Participants’ long-term memory was indexed on the basis of difference in their ability to recall separate ideas expressed in the Sun vignette at Times 1 and 2; the mean decline in performance is reported for participants who solved and did not solve the problem at Time 2 in both interactivity conditions in Table 1. Memory decline was constant across participants in both conditions and as a function of having solved the problem. A 2(solved, did not solve) × 2(low, high interactivity) between subjects’ ANOVA confirmed those impressions: the main effects of having solved the problem, *F* < 1, and interactivity, *F* < 1, were not significant, nor was the interaction, *F*(1, 59) = 1.54, *p* = .220.

Participants’ working memory capacity as gauged by their performance on a computation span test is reported in Table 1. Participants who solved the problem generally had higher C-Span scores than those who did not solve the problem, and this pattern was observed in both interactivity conditions. In a 2 × 2 between-subjects ANOVA, those who solved the problem (*M* = 21.3, *SD* = 9.0) scored significantly higher on the C-Span than those who did not (*M* = 14.1, *SD* = 8.0), *F*(1, 59) = 6.84, *p* = .011, but working memory capacity did not differ between participants in the high-interactivity condition (*M* = 20.7, *SD* = 10.3) and low-interactivity condition (*M* = 13.3, *SD* = 8.9), *F*(1, 59) = 1.64, *p* = .206; the interaction was not significant, *F* < 1.

## Experiment 1: discussion

Experiment 1 sought to explore the impact of interactivity on the ability to solve the CNP as well as the degree of incubation manifested in performance at Time 2 as a function of the level of interactivity. The CNP is a difficult problem, and the solution rates observed here are similar to those reported previously (e.g. Fioratou & Cowley, 2009; Fioratou, Flin, & Glavin, 2010; Silveira, 1971). Upon first encounter in the low-interactivity condition, only two participants could find the solution. This low solution rate is in part explained by how quickly participants gave up on the task: Generally, participants were less diligent in the low-interactivity condition.

Performance on the CNP was significantly better when participants could use and manipulate tangible chains in the high-interactivity condition than when restricted to just a pen and paper to find the solution. Higher working memory capacity, however, was associated with higher solution rates in both conditions, a finding that lends support to the role of working memory in insight problem solving.

More participants completed the CNP at Time 2, and those participants who completed the task successfully on both attempts were quicker to find the solution during their second attempt. As we had anticipated, the incubation effect was distinctly stronger in the high-interactivity condition: participants who did not solve the problem at Time 1 in the high-interactivity condition were more likely to solve the problem at Time 2 than the unsuccessful participants in the low-interactivity condition at Time 1. Thus, incubation effects were more clearly manifested in a high-interactivity context. In addition, performance at Time 2 could not be explained in terms of better long-term memory; memory decline was similar in both conditions.

We assumed higher levels of interactivity in the initial context would provide multiple opportunities for unplanned interactions, thus resulting in a richer, more dynamic initial representation of the task in terms of possible moves and possible paths to solution. However, it remains to be established whether the greater increase in solution rates at Time 2 with the high-interactivity version of the CNP is driven by restructuration through incubation on a richer, more dynamic representation of the task fostered at Time 1 (an incubation-driven performance improvement) or by restructuration through enactment on the high-interactivity version of the CNP at Time 2 (an enactment-driven performance improvement). If the richness of the initial representation is indeed key to the incubation effect, performance after incubation should therefore remain high when people work on the high-interactivity version at Time 1, and are presented with the paper-and-pencil version at Time 2. Conversely, when presented with the paper-and-pencil at Time 1, the resulting initial representation should be less amenable to restructuration and thus offer limited advantage to improve performance at Time 2. There is an alternative possibility, however: it may be that incubation effects are realised in situ. If this is the case, we should expect that working within a context with limited action possibilities at Time 2 (i.e. using the paper-and-pencil CNP version) would impede performance improvement even if reasoners were able to interact with a high-interactivity version at Time 1. Conversely, a context replete with action possibilities (i.e. using the high-interactivity CNP version) at Time 2 should allow performance to leap up. These conflicting predictions are summarised in Table 2 and were tested in Experiment 2.

**Table 2:** Incubation-driven vs. enactment-driven expected performance rise at Time 2

Initial interactivity level	Final interactivity level	
	Low	High
Incubation-driven performance improvement at Time 2		
Low	+^a^	+^b1^
High	++^b1^	++^a^
Enactment-driven performance improvement at Time 2		
Low	+^a^	++^b2^
High	+^b2^	++^a^

Time 1 = initial interactivity level, Time 2 = final interactivity level

^a^Denotes small (+) or large (++) increase in performance observed in Experiment 1

^b^Denotes small (+) or large (++) increase in performance expected in Experiment 2, under the (1) incubation-driven or the (2) enactment-driven hypothesis, respectively

## Experiment 2: method

### Participants

Sixty-one undergraduate and postgraduate psychology students (56 females) were recruited to participate in exchange for course credits (*M*_age_ = 21.43, *SD* = 4.21). All participants were naïve to the CNP prior to participation.

### Materials

The same materials from Experiment 1 were used.

### Design and procedure

Experiment 2 employed a 2 × 2 mixed design, with time as a repeated measures factor and interactivity level switch as a between subjects factor (low interactivity at Time 1 switched to high interactivity at Time 2 vs. high interactivity at Time 1 switched to low interactivity at Time 2). A similar procedure as that of Experiment 1 was employed aside from one key change: the interactivity level switch for participants at Time 2.

## Experiment 2: results

### CNP performance

#### Solution rates

As in Experiment 1, successful performance was classified using Silveira’s (1971) solution criterion. Solution rates are reported in Table 3: Fourteen (or 41%) of the participants solved the problem in the high-interactivity condition at Time 1, while four (or 15%) solved the problem in the low-interactivity condition; the difference in solution rates was significant, *χ*^2^(1, *N* = 55) = 5.03, *p* = .025; this pattern of solution rates replicates the pattern observed at Time 1 in Experiment 1. At Time 2, all 14 participants who solved the problem in the high interactivity at Time 1 solved the problem in the low-interactivity condition. Among the remaining 20 participants who had not solved it at Time 1 in the high-interactivity condition, only 1 (or 5%) solved the problem in the low-interactivity condition at Time 2 All four participants who solved the problem in the low interactivity at Time 1 solved it in the high-interactivity condition at Time 2; of the remaining 23 who did not solve the problem in the low-interactivity condition at Time 1, 14 (or 61%) now solved the problem when they switched to the high-interactivity condition at Time 2.

**Table 3:** Solution frequencies in the low- and high-interactivity conditions at Time 1 and 2, along with solution latencies, long-term memory performance (LTM; indexed as the difference in recall accuracy at Time 2), and working memory capacity (C-Span)

Time 1	Low interactivity		High interactivity	
	Yes	No	Yes	No
Freq	4	23	14	20
%	15%	85%	41%	59%
Latency to solution				
*M*	755.5		1107.6	
*SD*	306.6		408.6	

Time 2	High interactivity				Low interactivity			
	Yes	No	Yes	No	Yes	No	Yes	No
Freq	4	0	14	9	14	0	1	19
%	100%	0%	61%	39%	100%	0%	5%	95%
Latency to solution								
*M*	278.8		810.2		196.9		1094.0	
*SD*	148.1		326.1		153.6			
LTM index								
*M*	− 2.5		− 3.6	− 4.6	− 3.7		− 2.0	− 4.9
*SD*	1.3		1.5	1.3	2.2			3.3
C-Span								
*M*	23.0		25.7	23.3	19.4		20.0	16.6
*SD*	10.2		10.2	10.0	7.6			29.0

The switch in the level of interactivity substantially affected performance. This is particularly clearly revealed on the basis of cross experiment comparisons. Thus, of the participants who did not solve the CNP in a low-interactivity condition in Experiment 1 at Time 1, 5 (or 15%) solved the same version of the problem at Time 2. In Experiment 2, of the participants who did not solve the low-interactivity version of the problem at Time 1, 14 (or 61%) subsequently solved the problem in the high-interactivity condition at Time 2. Thus, incubation following the paper-and-pencil task (low interactivity) led to significantly higher rate of solution in a highly interactive realisation context, *χ*^2^(1, *N* = 56) = 12.64, *p* < .001. Conversely, the switch to a low-interactivity condition for participants who started in a high-interactivity condition depressed performance considerably. Of the 20 participants who failed to solve the problem at Time 1 in Experiment 2 in the high-interactivity condition, only 1 (or 5%) solved it at Time 2 in the low-interactivity condition. In Experiment 1, of the 16 participants who had not solved the problem at Time 1 in the high-interactivity condition, 7 (or 44%) solved it at Time 2. In other words, incubation following the high-interactivity task version resulted in a significantly lower rate of solution in the “paper-and-pencil” low-interactivity realisation context, *χ*^2^(1, *N* = 36) = 7.22, *p* = .007. Because two of the four cells of the contingency table had expected frequencies lower than 5, we also conducted a Fisher exact test, which was significant, *p* = .012, and supported the initial chi square test. Taken together, these results support the enactment-driven performance hypothesis (see Table 2), since, as Table 4 illustrates, switching from a high to a low level of interactivity resulted in a much smaller increase in performance than switching from a low-interactivity to a high-interactivity level.

**Table 4:** Performance improvement in Experiments 1 and 2 following incubation as a function of the initial (Time 1) and final (Time 2) level of interactivity

Initial interactivity level	Final interactivity level			
	Low		High	
	*M*	SE	*M*	SE
Low	+ 14%	0.03	+ 52%	0.04
High	+ 3%	0.03	+ 25%	0.04

#### Solution latencies

Latency to solution were lower at Time 2 compared to Time 1 for those participants who had solved the problem at Time 1 (see Table 3). In a 2 × 2 mixed ANOVA, the main effect of time was significant, *F*(1, 16) = 38.7, *p* < .001, but neither the main effect of order (high to low or low to high), *F* < 1, nor the interaction, *F*(1, 16) = 3.78, *p* = .07, was significant. Looking at how quickly participants solved the problem at Time 2, for those who had not solved the problem at Time 1, the analysis could only be conducted for participants in the low- to high-interactivity condition, since only one participant in the high to low condition solved the problem at Time 2. In the low to high condition, Time 2 successful participants were not faster than those who had solved the problem at Time 1, *t*(16) = − 0.299, *p* = .769. There was no difference in the level of perseverance among participants who did not solve the problem at Time 1 in the high-interactivity condition (*M* = 693 s, *SD* = 464) and in the low-interactivity (*M* = 716 s, *SD* = 386) condition, *t*(33) = 0.16, *p* = .875.

### Long-term and working memory performance

Participants’ long-term memory was indexed on the basis of the difference in their ability to recall separate ideas expressed in the Sun vignette at Times 1 and 2. The mean decline in performance for participants who solved, and did not solve, the problem at Time 2 in both interactivity conditions is reported in Table 3. Pooling the data across interactivity conditions, memory decline was a little less steep among participants who solved the problem (*M* = − 3.45, *SD* = 1.76) than among those who did not solve the problem (*M* = − 4.85, *SD* = 2.85). In a 2(solved, did not solve) × 2(low to high, high to low) between subjects ANOVA, the main effect of having solved the problem, *F*(1, 56) = 4.22, *p* = .045, was significant; however, memory scores did not differ between experimental conditions, *F* < 1; the interaction between success and experimental condition was not significant, *F* < 1.

Participants’ working memory capacity as gauged by their performance on a computation span test is reported in Table 3. Pooling the data across conditions, participants who solved the problem generally had higher C-Span scores (*M* = 22.52, *SD* = 9.21) than those who did not solve the problem (*M* = 18.85, *SD* = 24.94). However, in a 2(solved, did not solve it) × 2(low to high, high to low) between subjects ANOVA, the main effect of solving the problem was not significant, *F* < 1, nor was the main effect of the experimental condition, *F*(1, 56) = 1.63, *p* = .206; the interaction was also not significant, *F* < 1.

## Experiment 2: discussion

In Experiment 2 participants switched from a low- to a high-interactivity context or vice versa when they encountered the problem a second time 2 weeks later. At Time 1, participants were much more likely to solve the problem in the high- than the low-interactivity condition, replicating the pattern observed in Experiment 1. The effect of incubation was strongest when it was realised in a high-interactivity context. That is, the solution rate among participants who first encountered the CNP in a low-interactivity condition to then tackle the problem in the high-interactivity condition was significantly higher than when participants tackled the problem in a low-interactivity condition after initially working on the problem in a high-interactivity condition. This suggests that performance at Time 2 is primarily driven by the context of enactment rather than by the nature of the problem representation achieved prior to incubation. In addition, diligence among unsuccessful participants at Time 1 in Experiment 2 did not differ significantly between the low- and high-interactivity condition. Pooling the data across the interactivity conditions, participants who scored better on the long-term memory task were more likely to solve the problem at Time 2, suggesting that long-term memory might be implicated in moderating the learning effect across exposure to the problem; however, working memory capacity as measured with a computation span test did not differ between participants who solved and did not solve the problem at Time 2.

## General discussion

The present experiments investigated incubation and interactivity in problem solving using the CNP. In Experiment 1, participants reattempted the task with the same level of interactivity as with their initial encounter. In Experiment 2, participants reattempted the task with a different scope of action possibilities; they switched from low interactivity to high interactivity or high interactivity to low interactivity. As expected, in both Experiment 1 and Experiment 2, participants in the high-interactivity conditions solved the problem more frequently than those in the low interactivity at Time 1. Informed by the Systemic Thinking Model (Vallée-Tourangeau & Vallée-Tourangeau, 2017), we predicted that incubation would differ markedly as a function of the level of interactivity. This is indeed what was observed in Experiment 1. Experiment 2 enabled us to refine this finding by demonstrating that higher levels of interactivity also play a critical role in facilitating solution following incubation, in the realisation context. All participants who interacted with the high-interactivity version of the CNP were able to transfer their solution to the low-interactivity version. Meanwhile, participants who did not solve the problem in the initial context using a paper-and-pencil version were able to overcome their impasse when they could manipulate chains following a 2-week break. However, for those who did not overcome their impasse at Time 1 while working on a high-interactivity version of the CNP, incubation on a presumably richer representation of the problem elements did not facilitate insight when their thinking was later constrained in a deductive processing loop while working on a paper-and-pencil version at Time 2 This finding suggests that task realism or “concreteness” cannot account for the effect of interactivity, since having encountered the more concrete high-interactivity version of the CNP at Time 1 was not sufficient to promote success regardless of the interactivity level of the task at Time 2. This is an important finding which suggests that restructuring following impasse is not a purely representational process; the unique SysTM perspective paves the way for moving beyond comparing and contrasting predictions from purely internalist accounts, such as those from representational change theory or the criterion of satisfactory progress hypothesis, to consider how increasing interactivity and, by way of consequence, increasing reasoners’ opportunities to process perceptual inputs through physical manipulation of the problem elements plays a critical role in the enactment of insight.

Future research may further distil this finding by examining more closely the nature of the representations created by the low-interactivity and high-interactivity versions of the CNP and their actual role in overcoming the impasse. The SysTM assumes that inductive processing results from unplanned actions driven by the perception of affordances. Therefore, we purposely did not use verbal protocols to avoid artificially forcing participants to engage in a deductive processing loop where they would actively plan each of their moves instead of being guided by the action possibilities they perceived. In other words, SysTM would predict that instructing participants to narrate their concurrent thought processes might reduce performance under high levels of interactivity as this would encourage more deductive processing, and limit or constraint opportunistic insights emerging from unplanned actions triggered by the perception of affordances.

Likewise, SysTM would predict that a passive observation of physical movements (e.g. where a participant is observing another participant who attempts to solve the task in a high-interactivity context) would not be as successful, since this would presumably increase the load of deliberative thinking as the observer attemps to uncover the intentions of the actor’s moves.

Admittedly higher degrees of interactivity engage a number of different physical and cognitive processes. The relatively blunt measure of problem-solving success reported in these experiments does not in itself identify their relative contributions. Future research may also explore whether, and by which processes, higher interactivity fosters unplanned actions and richer task representations through a more refined analysis of action possibilities and their impact on cognitive processes (see, e.g. Steffensen et al., 2016 for a probatonic analysis of another insight task, the 17 animals problem).

The results in the present study were in line with other results showing that interaction with artefacts enhances problem-solving success (Fioratou & Cowley, 2009; Vallée-Tourangeau, et al., 2015; Vallée-Tourangeau et al., 2016b). The performance in the present experiments were considerably higher than those reported by Fioratou and Cowley (2009). This difference may be explained by the length of time permitted for participants to complete the task. Fioratou and Cowley allowed a maximum of 10 min, while participants in the experiments reported here could work on the problem for 30 min during each of the two testing sessions. Nonetheless, both the results of the present experiments and Fioratou and Cowley underscore the importance of working with a highly interactive version of the problem.

If an incubation effect is in part driven by participants adopting a fresh perspective on the problem, we would expect long-term memory scores to be higher for those who did not solve the problems at Time 2; however, if anything long-term memory scores were slightly higher among those who solved the problem at Time 2. In turn, while working memory capacity has been implicated in solving insight problems (e.g. Chuderski, 2014), the evidence is mixed (e.g. DeCaro, Van Stockum, & Wieth, 2016), and more important, previous work that explored problem solving in a high-interactivity task environment found no association between working memory scores and solution rates with a so-called insight problem (e.g. Vallée-Tourangeau et al., 2016b).

The CNP remains a difficult problem to solve due to the non-obvious solution required, namely breaking one of the chains into its separate components to use as bridging links to create the full necklace. Working with a highy interactive version of the problem facilitates discovering the solution. Walking away from the problem is also a good thing. We observed here that participants who did not solve the problem at Time 1 were more likely to solve it at Time 2. The novel and original findings reported here indicate that an effect of incubation was most clearly present when participants worked on the problem in a high-interactivity condition at Time 2. These findings highlight the importance of examining problem solving in different task environments as well as gauging incubation as a function of the scope of action possibilities afforded by the level of interactivity. Interactivity matters and helps participants recognise more quickly a path to solution when they previously failed to solve the problem.
